# The Central Midwives' Board

**Published:** 1902-03-08

**Authors:** 


### The Central Midwives' Board.

Under clause 4 of the Midwives Bill, which passed the second reading on the 26th ultimo, it is provided that the Central Midwives' Board shall consist of four registered medical practitioners, one of whom will be appointed by the Royal Colleges of Physicians and Surgeons and by the Society of Apothecaries respectively, and one by the Incorporated Midwives' Institute; two persons, one of whom shall be a woman, will be appointed by the Lord President of the Council, and one person by the Association of County Councils. Political and other organisations of women workers all over the country have maintained that, although the present Bill is an improvement on the one submitted to the House of Commons in 1900, it still is inadequate as to the representation of women on the proposed Midwives' Board. They declare that it provides inadequate representation of the one body at present representing the trained midwives, the Incorporated Midwives' Institute; that the exclusion of women from the local supervising authority, County Council, or County Borough Council, of which bodies no woman is permitted to be a member, and of want of security for the representation of women on the committees or District Councils or committees of such District Councils, to which the work of supervision may be delegated, is a serious injustice. They point out that women are at present eligible to serve on technical instruction committees, though the practice of appointing them on such bodies is by no means universal, and it may be presumed therefore that they will be excluded from the duty of supervising midwives locally unless their appointment be made compulsory by the Act.

There can be no doubt that women have the strongest claim to be fully represented upon all bodies which may be appointed by the Midwives Act to supervise midwives throughout the country. The medical profession is divided on the question of this Bill, and a majority of the profession have declared themselves as opposed to it, mainly on the ground that it will set up an inferior order of medical practitioner. We do not share this objection, and there are a large body of the more intelligent members of the profession who believe that the registration of midwives is necessary and should be made compulsory without delay. The Bill as it stands is the result of many years' discussion and labour; it represents the line of least resistance, and we are glad therefore that the Home Secretary should have expressed his hope that it may become law this year. We think on the whole the constitution of the Central Midwives' Board under Clause 4 of the Bill fairly represents all shades of opinion, and that it should prove in practice a good working body. The fact that one of the four registered medical members will be appointed by the Incorporated Midwives' Institute gives the midwives just that form of representation which is best calculated to enable them to express adequately their wishes and views in the matter.

We understand that at the last moment, although the direct representation of midwives through the Incorporated Midwives' Institute has been approved by the Committee on Law to which previous Midwives Bills have been sent, an attempt will be made to deprive the institute of its power of appointment. This would be a grave injustice in view of the fact that the Midwives' Institute is incorporated by Act of Parliament, that it has been in existence for 20 years, that its members represent at least one-seventh of the reputable midwives known to be working in the United Kingdom, that it has provided the funds and actively promoted the movement which has resulted in the present Midwives Bill, and that its council and officers are representative of all that experience proves to be best and most intelligent in this country so far as the training and improvement of midwives is concerned. The ground upon which an attempt is to be made to deprive the Midwives' Institute of their representation is that it is a purely voluntary association, not incorporated by Royal Charter. We can hardly suppose that any member of the House of Commons will seriously contend that incorporation by Act of Parliament is less effective or honourable than incorporation by Royal Charter, in which case the objection must fail on its merits. Divided as the medical profession is, and seeing that in no other way can a guarantee be given that the medical opinion in favour of legislation for midwives can be certainly secured on the Central Midwives' Board, except by conferring the power of appointing one medical practitioner on the Incorporated Midwives' Institute, we hope that section 1 of clause 4 will be allowed to stand unaltered. Unless the Incorporated Midwives' Institute has the statutory right to appoint at least one member of the Board, the midwives cannot be represented, and the Act must fail because Parliament will then be legislating for a particular class, whilst it deprives that class of all representation whereby alone it can protect its interests and promote the well-being of the whole body of women workers affected by the Act.

